# Administrative law as a determinant of public health

**DOI:** 10.17269/s41997-023-00826-w

**Published:** 2023-11-06

**Authors:** Stephen Thomson

**Affiliations:** ANU College of Law, The Australian National University, Canberra, Australia

**Keywords:** Public health, Global health, Determinants of health, Regulation, Governance, Santé publique, santé mondiale, déterminants de la santé, réglementation, gouvernance

## Abstract

Administrative law comprises the rules, values, and processes by which government and regulatory decision-making is subject to administrative monitoring, review, and accountability. It impacts public health in two ways: through the design, powers, and processes of institutions that enforce administrative law; and through the substantive rules of administrative law. Yet despite its fundamental regulation of the way in which public health decisions are made, insufficient research has been conducted on administrative law as a determinant of public health. Administrative law and public health operate as siloed academic disciplines with very little cross-disciplinary collaboration, engagement, or understanding. This results in major, untapped research opportunities exploring how administrative law could contribute to an optimized model of planetary health in both higher income and lower-middle income countries. Put simply, a holistic, global view of the determinants of public health must take due account of the accountability rules and controls that regulate how public health, and other, decisions are made. This commentary is a call to action to better understand how administrative law mechanisms, such as judicial review, administrative tribunals, ombudsmen, information commissioners, public auditors, and human rights monitors, can be designed or redesigned to better promote sustainable public health outcomes.

## Résumé

Le droit administratif comprend les règles, les valeurs et les processus qui assujettissent la prise de décisions gouvernementales et réglementaires à la responsabilité, aux examens et aux suivis administratifs. Il influence la santé publique de deux façons : par la conception, les pouvoirs et les processus des institutions qui appliquent le droit administratif, et par les règles de fond du droit administratif. Pourtant, bien qu’il régisse fondamentalement la façon dont les décisions de santé publique se prennent, il n’y a pas suffisamment d’études sur le droit administratif en tant que déterminant de la santé publique. Le droit administratif et la santé publique sont exercés en tant que disciplines universitaires cloisonnées, avec très peu de collaboration, de participation ou de compréhension entre elles. Il y a donc d’immenses possibilités de recherche inexplorées pour savoir comment le droit administratif pourrait contribuer à un modèle de santé planétaire optimisé, dans les pays à revenu élevé comme dans les pays à revenu intermédiaire ou faible. En clair, une perspective holistique et mondiale des déterminants de la santé publique doit tenir compte des règles et des contrôles de responsabilité qui régissent la prise des décisions de santé publique, entre autres. Notre commentaire est un appel à mieux comprendre comment les mécanismes du droit administratif, comme le contrôle judiciaire, les tribunaux administratifs, les protecteurs du citoyen, les commissaires à l’information, les auditeurs du secteur public et les observateurs ayant pour fonction de veiller au respect des droits de la personne, peuvent être conçus ou redéfinis pour favoriser des effets durables sur le plan de la santé publique.

## Introduction

Public health is impacted by a range of systemic factors including social (Braveman & Gottlieb, 2014), political (Kickbusch, 2015), and constitutional (Ip & Lee, 2021) determinants. Law has been recognized as a key determinant of health and to exert a powerful force on all social determinants of health (Gostin et al., 2019). Yet law is a vast discipline, and its various components impact public health in different ways. Medical law and tort law regulate clinical negligence and medical malpractice, impacting the delivery of healthcare. Environmental law regulates human interactions with the environment, impacting the health effects of pollution and climate change. Constitutional law regulates the limits of institutional authority, prohibiting acts and laws that violate health-protecting provisions in constitutional codes, charters, and human rights instruments. However, a key piece of this jigsaw is underrepresented in the literature on the determinants of health: administrative law.

Administrative law regulates how decisions, often at a granular level, are made by public bodies such as government departments, executive agencies, and regulators, including public health decisions and those impacting public health in other ways. Administrative law exists in every legal system and is found at the national and sub-national levels in both federal and non-federal systems. It is mainly found in legislation and court judgements. But how, specifically, might administrative law impact public health? There are two aspects to this question.

## Design, powers, and processes of administrative law institutions

The first concerns the design, powers, and processes of the institutions that enforce, and in some instances create, administrative law: courts (in judicial review), administrative tribunals, ombudsmen, information commissioners, public auditors, and human rights monitors, to name some of the main examples. Are public health outcomes more or less likely to be promoted by tribunal review conducted by the executive branch (as in Australia and the United States) or the judicial branch (as in the United Kingdom)? Are they more or less likely to be promoted by ombudsmen that can (as in several African countries) or cannot (as in most other countries) award directly enforceable remedies? Are desirable public health outcomes better supported by creating specialized health ombudsmen (as in Brazil) or generalist ombudsmen (as in Mexico)? Are they best promoted by judicial review procedures that tend to allow, or restrict, challenges brought by public interest groups against public health authorities? Do strong (as in Canada) or weak (as in China) freedom of information and whistleblowing frameworks better facilitate desirable public health outcomes? What about systems that tend to limit or expand administrative discretion?

We do not have focused answers to these questions. For example, the Australian Administrative Appeals Tribunal (AAT)—a major tribunal conducting independent review of federal government decisions—is in the process of abolition, with a new tribunal soon to be established. The AAT hears appeals against various health-related decisions including social welfare payment, worker health and safety, and therapeutic goods decisions. But are the powers, rules, and procedures of its replacement being designed in a way most likely to optimize public health outcomes? Unsurprisingly given the lack of research in this field, the question does not appear to have been asked, let alone answered (Attorney-General’s Department, 2023)—institutional design is often guided by administrative efficiency and operability considerations rather than empirically driven, evidence-based principles of institutional design and their causal relationship with substantive policy outcomes (though, as policy objectives can change, this is not a wholly unmeritorious approach). The matter is further complicated by the fact that the AAT hears a wide range of appeals against decisions made under more than 400 pieces of federal legislation, including those that have little to do with public health. Does this favour the creation of a specialist health tribunal rather than investing appellate powers against health decisions in a generalist tribunal?

Yet most, if not all, AAT decisions—not just health-specific decisions—may impact public health in direct or indirect ways. How could AAT (or for that matter any tribunal, internal review, or other administrative law) process be designed and operated so as to more positively affect the mental health of their users (Clemente & Padilla-Racero, 2020)? Do the processes for challenging decisions promote feelings of inequity and powerlessness? Do they entrench and perpetuate socio-economic inequalities with their consequential effects on health (Komro et al., 2013)? Are processes for challenging (for example) social housing decisions too complex, non-transparent, expensive, or time-consuming, such that individuals are likely to stay for longer in housing that negatively affects physical and/or mental health? And given administrative law’s emphasis on process, to what extent is there implementation fidelity (Fixsen et al., 2005)?

## The substantive rules of administrative law

The second way in which administrative law impacts public health is not a matter of institutional design, but the substantive rules of administrative law. Their starting point is what a decision-maker is permitted to do, with an emphasis on the limits of discretionary authority. That is usually determined by reference to legislation in the first instance—legislation which sets out the decision-maker’s powers and the processes by which they may be exercised—with other rules, including judge-made law, built on that foundation. While administrative law varies between countries (Cane et al., 2021), there are rules commonly encountered across legal systems. These include specific procedural and substantive requirements with which decision-makers (including public health decision-makers) must comply: for example, impartiality, good faith, no predetermined outcome, consultation with relevant stakeholders, absence of legal error, support by cogent reasons, due account of human rights, and substantive reasonableness or proportionality. Vague as those may sound to a non-legal audience, administrative law contains complex, detailed provisions on these rules (Wade et al., 2022). A decision-maker in non-compliance with these requirements may have their decision investigated by an ombudsman, reversed by a tribunal or invalidated by a court. For this reason, administrative law is regarded as an important facet of public sector accountability and the so-called administrative justice system (Hertogh et al., 2022).

The rules of administrative law apply not only to public health decision-makers, but also to decision-makers in (almost) all areas of government and administration, such as licensing, taxation, and urban planning. Evaluating the effectiveness of public health interventions on public health is clearly a worthy subject of investigation, falling within the scope of the classical definition of ‘public health law’ given by Gostin (Gostin, 2000). But the focus here is broader—more in the mould of ‘public health law research’ defined by Burris et al. as ‘the scientific study of the relation of law and legal practices to population health’ (Burris et al., 2010). This recognizes that law can impact public health even when it is not specifically a law embodying a public health intervention. For example, some decision-makers are required by administrative law to give to individuals reasons for their decisions (again, the extent of this varies by country). Reasons may reduce conflict, tension, and stress, with positive consequences for physical and mental health; ‘therapeutic’ models of complaints resolution have even been proposed in an administrative law context (Williams et al., 2022). Which bodies are required to give reasons and in what circumstances may therefore have direct health implications.

Nevertheless, the stakes for public health may be heightened where administrative law is applied directly to a public health intervention. The administrative law requirement that ‘improper purposes’ must not guide a decision-making process means that a health authority’s decision to impose lockdown and quarantine regulations to control the transmission of a communicable disease could not lawfully have economic or social purposes at its core. Similarly, the rule on ‘legitimate expectations’ means that a pharmaceutical regulator’s decision to remove the availability of a medicine from existing patients may be unlawful. Administrative law can be a matter of life and death: the English Court of Appeal held in the famous case of *R v Cambridge Health Authority, ex parte B* [1995] 2 All ER 129 that a public health authority’s decision did not violate the rules of administrative law when it refused to fund a second bone marrow transplant for a child who had suffered a relapse of acute myeloid leukaemia.

## The potential of administrative law for improved public health

Administrative law therefore has the potential to promote public administration and ‘good governance’ values such as evidence-based decision-making, consistency, rationality, open-mindedness, transparency, due process, fairness, and efficiency (Daly, 2016). These are promoted both by the content of the substantive rules of administrative law and the design, structure, and culture of the institutions that enforce them. A public health audience will readily appreciate how these values can support public health decision-making, facilitating open, transparent, scientifically grounded, and methodologically robust public health decisions (Kinney, 2022). But the empirical relationship between individual values promoted by administrative law is likely multi-layered and complex, as some of these values in isolation—for example, efficiency—might offer limited potential for desirable public health outcomes. Efficiency might characterize public health decision-making in an authoritarian system with poor human rights standards, but research already indicates that decision-making in such systems may be more likely to produce degraded public health outcomes (Kavanagh, 2020). Other values, such as transparency, are likely to materially contribute to desirable public health outcomes (O’Malley et al., 2009).

Existing research barely scratches the surface of the extensive research questions at play in this interdisciplinary space (Mello & Zeiler, 2008). That is primarily because administrative law and public health are siloed academic disciplines with very little cross-disciplinary collaboration, engagement, or understanding. It is certainly not due to any lack of research opportunities or the great potential for improved planetary health that inheres in such research questions. As public health crises loom worldwide—from antibiotic resistance to the obesity epidemic—serious consideration must be given to the design (or redesign) of administrative law systems to best equip human societies to manage and mitigate their effects. Public health crises can have ruinous effects on scarce public resources, the natural environment, educational attainment, socio-economic improvement, economic productivity, and even national security (Police & Ruppert, 2022), so the global relevance and potential gains of such research are high. When law and public health operate in insufficiently integrated spaces, public health interventions can raise significant legal uncertainties (Thomson, 2022) and drastic consequences for rights protections (Thomson & Ip, 2020). As a judge of the High Court of Botswana and the Residual Special Court of Sierra Leone recently explained, the ‘law and justice sector plays a critical, though often unacknowledged, role in every health challenge’ (Dingake, 2017). This new angle of investigation is worthy of pursuit in its own right, as the wide range of administrative law controls and values do not map neatly onto the globally diverse governmental (e.g. democratic, authoritarian, hybrid) or constitutional (e.g. unitary, federal, devolved) system types. Some work has been conducted to investigate constitutional and political determinants, such as democracy, on health (Wise & Sainsbury, 2007), but a specifically administrative law‒focused investigation is lacking.

Desirable public health outcomes depend on a range of factors including constitutional factors (such as age and genetics), individual lifestyle, social and community networks, living and working conditions, and socio-economic, cultural, and environmental conditions (Dahlgren & Whitehead, 1991). Yet a holistic, global view of the determinants of public health must also take due account of the accountability rules and controls that regulate how public health, and other, decisions are made. Gostin et al. have argued that health laws must be supported by good governance (Gostin et al., 2019): administrative law, which is inherently normative, informs and reflects many of the values of good governance. Focused research on administrative law as a determinant of public health could contribute to an optimized model of planetary health in both higher income and lower-middle-income countries. Future research should, in particular, investigate which models of administrative review tend to correlate with improved public health outcomes at both the individual and community/population levels. Research should also investigate whether particular models of administrative review better promote desirable public health outcomes across the board or only in particular types of legal, political, and cultural system, as there might not be a ‘one size fits all’ model. Researchers, funding agencies, policymakers, and decision-makers should be astute to the extensive opportunities that administrative law offers to improve planetary health.

## Data Availability

Not applicable.
